# Intestine perforation by an accidental ingested SARS-CoV-2 nasopharyngeal swab; a case report

**DOI:** 10.1016/j.ijscr.2022.107378

**Published:** 2022-06-30

**Authors:** Yvonne Versluijs, Niels Keekstra, Fabian A. Holman

**Affiliations:** Department of Surgery, Leiden University Medical Center, Albinusdreef 2, 2333 ZA Leiden, the Netherlands

**Keywords:** Intestinal perforation, SARS-CoV-2, Exploratory surgery, Case report

## Abstract

**Introduction and importance:**

Gastrointestinal tract perforations as a result of foreign body ingestion are rare. Most ingested foreign bodies pass the intestines without complications. However, in 1 % of cases intestinal perforation occurs. We present the case of a 56-year old patient with an extensive surgical medical history who presented at the emergency department with progressive abdominal pain two weeks after accidental SARS-CoV-2 swab ingestion.

**Case presentation:**

On presentation patient was tachycardic and had generalized abdominal tenderness. A CT scan showed free intraperitoneal air and fatty infiltration of the ileocecal anastomosis (after an ileocoecal resection at the age of 46) continuing to the distal sigmoid. Emergency exploratory laparotomy revealed a covid swab in the abdominal cavity with an indurated area of the sigmoid without perforation. Post-operative care was uneventful, and patient was dismissed after four days.

**Clinical discussion:**

Due to his medical history and the fact he was advised to regularly self-test for COVID, he routinely performed an oropharyngeal swab. Unfortunately, this resulted in swallowing the swab. A perforation tends to happen in regions of acute angulation, such as an anastomosis. Although the CT scan suggested the perforation was at the ileocecal anastomosis, no perforation was found during surgery, while the swab was found loose in the peritoneal cavity.

**Conclusion:**

Initial treatment should focus on endoscopic removal. In the case of gasto-intestinal perforation, surgery becomes the treatment of choice. A foreign body can migrate to peritoneal cavity without peritonitis or visible perforation perioperative.

## Highlights

• To the authors best knowledge this is the first case of intestinal perforation after an ingested SARS-CoV-2 nasopharyngeal swab.
• A foreign body can migrate to peritoneal cavity without peritonitis or visible perforation perioperative.
• If endoscopy fails to remove it and there is a high risk of perforation, surgical removal should be considered before severe complications develop.
• In the case of gasto-intestinal perforation, surgery becomes the treatment of choice.

## 1 Introduction and importance

Ingestion of a foreign body is a common cause of admission to the emergency department [1], [2]. Although most of foreign bodies transit through the gastrointestinal tract (GI) without complications, intestinal perforation occurs in up to 1 % of cases [1], [3]. We report the rare case of a 56-year-old man with an extensive surgical medical history, who suffered an intestinal perforation, two weeks after accidental ingestion of a SARS-CoV-2 nasopharyngeal swab.

This case report is written in line with the SCARE 2020 criteria [4].

## 2 Case presentation

### 2.1 Past surgical history

The patient in this case report had an extensive surgical past history. At the age of 38, patient underwent laparoscopic gastric banding surgery for morbid obesity which was followed by an abdominoplasty two years later. At the age of 45, laparoscopic gastric bypass was performed. A year later he underwent surgery for a hernia cicatricalis and a laparoscopic cholecystectomy. Due to irritation of the colon by the gastric band catheter, an ileocecal resection was performed. This procedure was complicated by leakage of the lesser curvature, for what he underwent surgery to repair this leakage. At the age of 52, another correction hernia cicatricalis was performed. After that, he underwent surgical correction of a diaphragmatic hernia with a biologic mesh, which resulted in permanent right hemidiaphragm paralysis.

### 2.2 Clinical presentation

This 56-year-old male was referred by the general practitioner to the emergency department (ED) of an academic hospital, with progressive severe abdominal pain over the past 4 h, two weeks after accidental ingestion of a SARS-CoV-2 nasopharyngeal swab. During the Omicron period, people were advised by the Dutch Ministry of Health, Welfare and Sport to perform oropharyngeal tests in addition to nasal self-sampling, since there was evidence the sensitivity of the COVID swab would be improved [5]. Given his medical history, the patient was advised to perform regularly self-tests and reach deep in the pharynx. This resulted in an accidental ingestion for which patient had multiple consultations with his GP who expected an uneventful passage of the swab. At admission on the ED, the patient was afebrile and tachycardic with a pulse rate of 120/min. Physical examination showed diffuse abdominal pain with no clinical signs of generalized peritonitis. The laboratory parameters showed increased inflammatory markers (leukocytes: 17.7, c-reactive protein: 9) and further normal hematological, renal, and hepatic profiles. Subsequently, oral contrast-enhanced computed tomography demonstrated the presence of free intraperitoneal gas and fatty infiltrations around the ileocecal anastomosis with extension to the distal sigmoid colon. These radiological features suggested the diagnosis of intestinal perforation. The covid swab was not visible on the CT.

### 2.3 Management and outcome

**Fig. 1:**
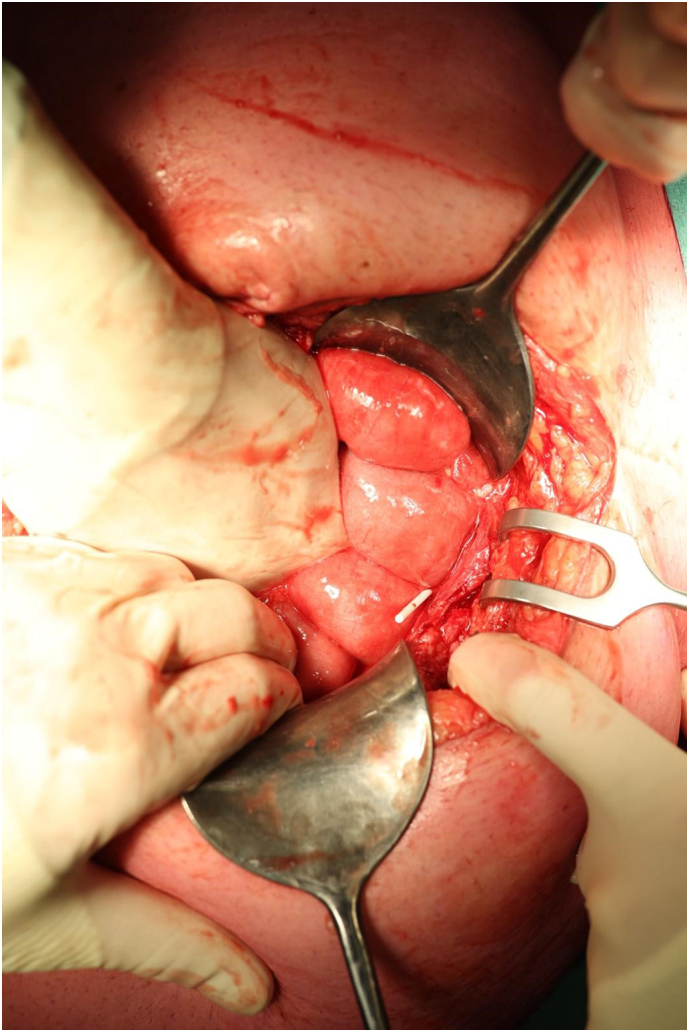
Abdominal exploration showing the covid swab in the free abdominal cavity.

Patient was admitted to the hospital and taken to the operating room for emergency exploratory surgery by an experienced abdominal surgeon. He was found to have purulent abdominal fluid, but no intestinal contents. The covid swab was found in toto in the free abdominal cavity (Fig. 1) and was removed. No abnormalities were found around the anastomosis of the ileocecal resection and rest of the small intestines. The sigmoid had a distinct indurated area; most likely the swab perforated through the wall and the perforation healed. No more perforations were observed during inspection. A surgical silicone drain was left in the abdominal cavity and was removed two days postoperative. In-hospital recovery was uneventful and he was discharged after 4 days. Four weeks after discharge patient developed wound dehiscence which was treated with wound dressings.

### 2.4 Clinical discussion

Incidental ingestion of a foreign body is a common problem [3], and it could be critical and fatal [6]. The majority of cases before 1900 were woman holding pins and needles between their lips, and nowadays it happens mainly among children and adults with psychiatric conditions [3], [7]. However, it can happen to anyone, as it happened to this patient. Due to his medical history and the fact he was advised to regularly self-test for COVID, he routinely performed an oropharyngeal swab. Unfortunately, this resulted in swallowing the swab. Complications of SARS-CoV-2 swab tests are described in previous literature but intestinal tract perforation has not been reported before [8]. To the authors best knowledge this is the first case of intestinal perforation after an ingested SARS-CoV-2 nasopharyngeal swab.

After ingestion, the characteristics of the object determine whether it can perforate, progress to an obstruction or leave the body without complications. The intestine protects itself from perforation due to intrinsic ability; the mucosa enlarges the bowel wall at the point of contact, which leads to easier pass of the foreign body [3]. Nearly 80 to 90 % of swallowed foreign bodies evacuate via the rectum among healthy patients [9]. A perforation tends to happen in regions of acute angulation, such as an anastomosis, or in areas near intestinal adhesions or resections [10]. Although the CT scan of our patient suggested the perforation was at the ileocecal anastomosis, no perforation was found during surgery, while the swab was found loose in the peritoneal cavity. Presumably the perforation had healed at the time of surgery since the swallowing happened two weeks before. In a case report of Kamali et al. [11], a patient was described with a foreign body in the lower abdomen and pelvis which migrated from the rectum to the peritoneal cavity without peritonitis.

If a foreign body is ingested and detected, the first approach should be endoscopic removal [7]. If endoscopy fails to remove it and there is a high risk of perforation, surgical removal should be considered before severe complications develop.

## 3 Conclusion

We present the case of a gastro-intestinal perforation as the result of ingestion of a SARS-CoV-2 nasopharyngeal swab. If endoscopy fails to remove it and there is a high risk of perforation, surgical removal should be considered before severe complications develop. In the case of gasto-intestinal perforation, surgery becomes the treatment of choice. A foreign body can migrate to peritoneal cavity without peritonitis or visible perforation perioperative.

## Sources of funding

No funding was required for this research paper.

## Ethical approval

This study is exempt from ethnical approval.

## Consent

Written informed consent was obtained from the patient for publication of this case report and accompanying images. A copy of the written consent is available for review by the Editor-in-Chief of this journal on request.

## Author contribution

Y. Versluijs (YV), Niels Keekstra (NK), Fabian A. Holman (FAH); study concept and writing the paper.

## Registration of research studies

N/a.

## Guarantor

Y. Versluijs.

## Declaration of competing interest

None.
